# Innovative strategies developed to mitigate the impact of COVID-19 pandemic on polio surveillance in Nigeria, 2020

**DOI:** 10.11604/pamj.supp.2023.45.2.38261

**Published:** 2023-09-07

**Authors:** Aboyowa Arayuwa Edukugho, Sume Gerald Etapelong, Saheed Gidado, Samuel Luka Abbott, Abdullahi Walla Hamisu, Isiaka Ayodeji Hassan, Kabir Yusuf Mawashi, Tesfaye Bedada Erbeto, Ndadilnasiya Endie Waziri, Patrick Nguku, Bolu Omotayo, Usman Said Adamu

**Affiliations:** 1 African Field Epidemiology Network, Federal Capital Territory (FCT), Abuja, Nigeria,; 2 World Health Organization, Country Office, Abuja, Nigeria,; 3 National Primary Healthcare Development Authority, Federal Capital Territory (FCT), Abuja, Nigeria,; 4 Centers for Disease Control and Prevention, Atlanta, Georgia, United States

**Keywords:** COVID-19 pandemic, Acute Flaccid Paralysis surveillance, surveillance officers, innovative, strategies, polio eradication

## Abstract

**Introduction:**

following the spread of the COVID-19 pandemic to Nigeria, the Federal Government of Nigeria restricted human and vehicular movements to curb the spread of the disease. This action had a negative impact on Acute Flaccid Paralysis (AFP) surveillance, with a resultant reduction in the number of AFP cases reported. This paper describes the impact of the COVID-19 pandemic on poliovirus surveillance in Nigeria and the proactive interventions by Nigeria´s polio program to mitigate the impact of COVID-19 on polio surveillance.

**Methods:**

nine innovative strategies were implemented in all 774 Local Government Areas (LGA) of the 36 states and Federal Capital Territory (FCT) of the country. These strategies were developed by the national surveillance officers and operationalized by sub-national surveillance officers with different strategies starting at different epidemiological weeks from week 14 to 23, 2020. Many of the strategy innovations were technology-based and included: the use of mobile phones to send the AFP case definition and video by WhatsApp or by SMS, the use of state-specific toll-free numbers and Mobile Telephone Network (MTN) (mobile service provider) CallerfeelTM to community informants (CI) who were the main targets of the interventions to increase case detection and reporting. Others included the use of abridged e-surveillance integrated supportive supervision (ISS) checklists, virtual monthly DSNO meetings, and batched AFP stool specimen transportation network.

**Results:**

compared to the same period in 2019, the cumulative rate of AFP case detection and reporting had gradually declined from 39.1% in January to 16.7% before the commencement of the interventions in week 20, 2020. However, the detection and reporting increased by 57.% from week 20 to week 47 compared to the same period in 2019. This is because with COVID-19, hospital visitation dropped, and the sick remained in the communities, so the CI network was relied on to detect and report AFP cases. The cumulative proportion of AFP cases reported by community informants as of week 47 increased from 13% in 2018 to 21% in 2020. This indicates an increase of 38%. Thirty-five AFP cases were detected and reported using the MTN Caller Feel strategy, while 15 cases were reported through state-specific toll-free numbers.

**Conclusion:**

the implementation of the innovative strategies was able to mitigate the low AFP case detection and reporting observed at the initial stage of the COVID-19 pandemic. The use of technology facilitated reaching the CI network, which was more instrumental in detecting and reporting the cases.

## Introduction

The Global Polio Eradication Initiative (GPEI) was inititated by World Health Assembly resolution in 1988 [1,2]. Global strategies for polio eradication include high levels of routine immunization, supplementary immunization activities, robust acute flaccid paralysis (AFP) surveillance and mop-up campaigns targeted to local areas with suspected or proven poliovirus transmission [3-5]. Acute flaccid paralysis (AFP) surveillance focuses activities where poliovirus transmission is found, allows for prompt detection in the case of an outbreak, and provides evidence of the lack of transmission. For any World Health Organization (WHO) region to be certified as polio-free, there must be no detection of poliovirus for 3 consecutive years, with high quality surveillance systems meeting performance indicators to detect poliovirus [1,6,7]. Therefore, poliovirus surveillance remains the backbone for GPEI and the primary means is AFP surveillance [5,8,9]. Systematic sampling of sewage water and testing for poiiovirus (envitonmental surveillance) can supplement AFP surveillance.

The two main performance indicators used to determine the sensitivity of the AFP surveillance system to detect the transmission of poliovirus are the non-polio AFP rate (NPAFP) and the proportion of AFPs case with specimens meeting stool adequacy criteria. For the standard NPAFP rate, at least 1 AFP case is detected that has been discarded as not being polio per 100,000 population aged < 15 years annually in non-endemic areas, while in endemic regions or those endemic prior to 2014, the target rate is 2 per 100,000 [9,10]. ´Adequate´ stool specimens are two stool specimens of sufficient quantity for laboratory analysis, collected at least 24 hours apart, within 14 days after the onset of paralysis, and arriving in the laboratory in good condition [9,10]. The global target of stool adequacy is 80% of specimens from AFP cases. Nigeria has continued to achieve these two indicators nationally and subnationionally [11]. The country´s complete documentation for wild poliovirus (WPV)-free status was accepted by the African Regional Certification Commission (ARCC) in June 2020. As Nigeria was the last country in the WHO Afircan Region with confirmed WPV transmission, the African Region was subsequently certified WPV-free in August 2020 by the ARCC. A key recommendation to Nigeria by the certification body was the need to sustain high quality surveillance performance in the country [12-14].

In December 2019, an outbreak of novel coronavirus disease (COVID-19) was reported in China [15]. The outbreak rapidly spread across international borders to many countries, including Nigeria. The disease was declared a pandemic by the World Health Organization (WHO) on March 11, 2020 [16,17]. The first case of COVID-19 in Nigeria was reported on February 27, 2020 in Ogun state in southwestern part of the country. Thereafter, the disease was identified across all the 36 states and FCT [18,19]. As part of measures to curtail the spread of COVID-19, the Government of Nigeria declared a total lockdown in March 2020 in Lagos, Ogun and FCT which were the most affected geographical territories at the time [20]. Similar restrictions of movement to varying degrees were imposed by other state Governments, while interstate travel restrictions were later imposed across the 36 states and FCT by the Federal Government to minimize the spread of COVID-19, especially from states with extensive community transmission [20].

The COVID-19-induced movement restrictions affected public health services and surveillance, including AFP surveillance. Active surveillance for AFP, case detection and investigation, AFP verification, sample transportation and surveillance supportive supervision reduced substantially in absolute terms. The frequency of environmental sample collection was reduced from weekly to fortnightly. Sample shipments were delayed because they had to be batched at designated points until transported to the respective laboratories in Ibadan, Oyo State and Maiduguri, Borno State. Scheduled trainings and routine meetings to boost and evaluate the performance of frontline surveillance officers could not be held. Consequently, the number of AFP cases detected and reported dropped by 63% by epi week 13 2020 compared to the same period in 2019 at the national level and to a varying extent across the 36 states and the FCT. A surveillance contingency plan within the context of COVID-19 pandemic was developed to guide surveillance officers on the conduct of poliovirus surveillance during this period. The development of a national contingency plan stems from the guidance provided by WHO [21]. We describe the impact of COVID-19 pandemic on poliovirus surveillance and the proactive interventions by Nigeria´s polio program to mitigate the impact of COVID-19 on surveillance.

## Methods

**Setting:** Nigeria is located in West Africa. With an estimated population of 211 million, it is the most populous country in Africa and the seventh-most populous country globally. Adminitratively, the country has 36 states and FCT. These states are grouped into six geopolitical zones: north central, northeast, northwest, southeast, south-south and southwest [22]. According to the 2005 census of health facilities conducted by the Federal Ministry of Health (FMOH), Nigeria has a total of 23,640 public and private hospitals. There are two national polio laboratories located in Maiduguri and Ibadan, northeastern and southwestern Nigeria respectively that are annually accredited by WHO. Both laboratories process stool and sewage samples from AFP cases and environmental sites, respectively [23].

**Development of contingency plan:** the national surveillance working group (NSWG) developed, planned, and monitored the contingency plan based on guidance by GPEI [21]. The NSWG is one of the working groups at the National Polio Emergency Operation Center (NEOC) that coordinates and monitors polio surveillance activities and interventions. The membership is drawn from the government and in-country GPEI partners. Coordination meetings of the working group are held weekly. Three versions were developed and shared in epi weeks 14, 18 and 30, of 2020, respectively. The plan contained the interventions with which to start, to continue, and to pause/stop considering the phase of the pandemic across the national and sub-national levels. Table 1 shows the innovative approaches for improving polio surveillance performance during the COVID-19 pandemic as implemented and guided by the contingency plan.

**Table 1:** innovative approaches for improving polio surveillance performance in the context of COVID-19, Nigeria 2020

Strategies	Initiation Epi Week
Surveillance contingency plan	Version 1: Week 14 Version 2: Week 18 Version 3: Week 30
Integrating AFP surveillance briefing with nationwide COVID–19 health worker sensitization	Week 18 – 22
State specific toll-free number	Week 20
Weekly AFP WhatsApp video messaging	Week 21
Abridged E-surveillance checklist	Week 21
Weekly SMS reminders to community Informants and surveillance focal persons	Week 22
Abridged ISS checklist	Week 23
DSNO virtual monthly meeting	Week 23
AFP stool specimen transportation Network	Week 23
MTN CallerFeel for polio surveillance	Week 24

Footnote: AFP: Acute Flaccid Paralysis; Epi: Epidemiology; E-Surveillance: Electronic surveillance; SMS: Short message system; ISS: Integrated supportive supervision; DSNO: Disease Surveillance and Notification Officer; MTN: A mobile network carrier in Nigeria

The following innovative approaches were included in the contingency plan and implemented for the first time in the country.

**Integrating AFP surveillance briefing with nationwide COVID-19 health worker sensitization:** in May 2020, the National Primary Health Care Development Agency (NPHCDA) organized a nationwide preparedness and response training on COVID-19 for frontline health workers to guide against transmission of the disease during health worker-client interaction. To enhance active surveillance for AFP, a 30-minute presentation on AFP surveillance to remind health workers on the need to continue to detect and report AFP cases was included in the training curriculum.

**State-specific polio surveillance toll-free numbers:** to enhance the report of suspected AFP cases by community members, state surveillance officers obtained toll-free numbers that community members could use to report suspected AFP cases. The toll-free number was obtained and used for both AFP and COVID-19 surveillance. Prior to the outbreak of COVID-19, there was no existing toll-free platform for reporting for AFP surveillance in the country.

**Weekly AFP WhatsApp video messaging:** auto-visual AFP detection and reporting (AVADAR) AFP case definition videos were shared through WhatsApp every week. Auto-visual AFP detection and reporting (AVADAR) videos, available in different languages, including English and some local dialects (Hausa, Igbo, Yoruba, and Pidgin), were shared by the NSWG with the state surveillance officers. The state surveillance officers then shared the videos to the Local Government Area (LGA) disease surveillance and notification officers (DSNOs) and Assistant DSNOs (ADSNOs), who in turn shared with the surveillance focal persons (SFPs) at health facilities (HFs) and community informants (CIs). A state monitoring tool was developed by NSWG and shared with the state surveillance team for weekly reporting.

**Abridged electronic surveillance (eSurve) checklist:** the eSurve checklist is mainly used by DSNOs and ADSNOs for active surveillance. The abridged eSurve checklist was developed to reduce the time spent by these surveillance officers during active case search at the HFs as a preventive measure to COVID-19. The existing 11-paged eSurve checklist, which had 49 questions, was revised to a shorter version with just 16 questions contained on three pages without losing the key indicators of quality surveillance supervision. The abridged eSurve checklist was also available as a hard copy version. The surveillance officers administered the abridged checklist physically at the HFs or remotely by making phone calls to the SFPs if they are unable to visit the facility.

**Weekly short message service (SMS) reminders:** short message service (SMS) reminders were sent to SFPs and CIs to remind them and raise awareness on the need to intensify AFP active case search and reporting to the programme through the established reporting channel. Before commencement, the state surveillance officers updated the database for SFPs and CIs and shared with the NSWG. Community informants´ names and mobile phone numbers totalling 82,091 and those of SFPs totalling 9,120 were line-listed across all the 36 states and the FCT. In addition, 390 Red Cross community volunteers working in three states (Jigawa, Katsina, and Zamfara) were line-listed. Altogether, 60,709 (74.0%) CIs, and 369 (94.6%) Red Cross community volunteers had SMS-enabled mobile phone numbers. Similarly, 8,828 (96.8%) SFPs had SMS-enabled mobile phone numbers. The NSWG then developed SMS reminder messages in English and subsequently translated them into Hausa, Igbo, Yoruba, and Pidgin as was done for the weekly AFP WhatsApp video messaging. The messages were then transmitted weekly from NSWG directly to all SFPs and CIs who had active mobile phone numbers as per the database shared by each state.

**Abridged integrated supportive supervision (ISS) checklist:** the ISS checklist is mainly used by implementing partners for supportive supervision. The abridged form was also developed to reduce the time spent by these officers while carrying out supportive supervision to SFPs at the HFs. The main ISS checklist was reviewed to shorten it from 69 questions contained in 13 pages to an abridged version with 30 questions contained in 9 pages. If the officer could not visit the facility, a separate ISS checklist was developed and applied remotely using a mobile phone. This checklist uses both the hard copy and Open Data Kit (ODK) version.

**Disease surveillance and notification officers virtual coordination meeting:** monthly coordination meetings with DSNOs are held to review surveillance performance at the LGA level with a view of closing any identified gaps. The contingency plan implemented since week 14, marking the commencement of total lockdown for the entire country, recommended that DSNO monthly meetings should not be held. Although most states stopped physical face-to-face monthly DSNO coordination meetings, others who had few LGAs continued to hold the meetings. But from week 23, funds were provided to all states to commence virtual coordination meetings with DSNOs, ADSNOs and environmental sample (ES) collectors.

**Figure 1:**
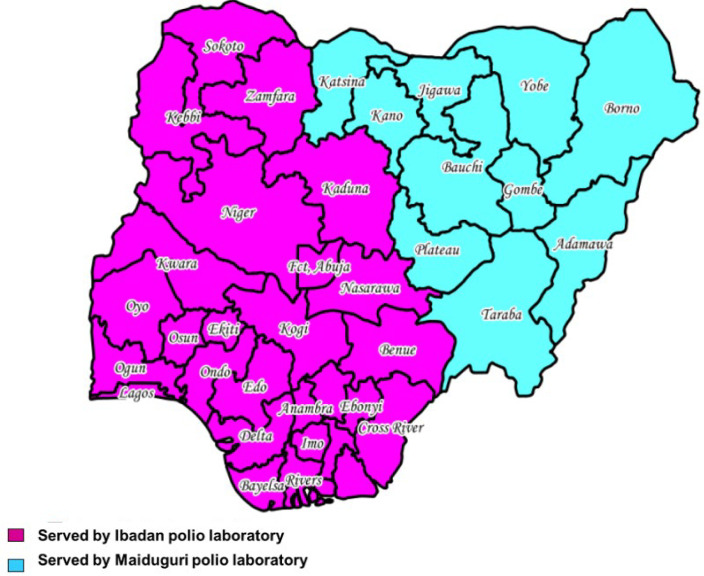
map of Nigeria showing states served by Ibadan and Maiduguri polio laboratories, Nigeria, December 2020

**Batching of polio specimen for transportation:** polio specimen from AFP and ES were experiencing delay from points of collection to the two polio laboratories located in Ibadan, Oyo State, south-western zone and Maiduguri, Borno State, north-eastern zone of the country owing to the nationwide lockdown. This delay affected the analysis of specimens and classification of specimens. To resolve this situation, specimen batching hubs were established at different points to cater for specimens coming in from the different zones of the country. At scheduled intervals, the batched specimens were then transported to designated laboratories by pre-identified transport couriers. The batching hub catered for states within the south-eastern and south-southern zones in Asaba, Delta State. While the Ibadan polio laboratory transport network recieved specimens from 15 routes; the Maiduguri polio laboratory had 5 routes. The advantage of this transportation arrangement is that few drivers on short distances were on the move each time, thereby shortening exposure time to SARS-CoV-2, reducing the risk of viral transmission. Figure 1 shows the map of Nigeria showing states served by the two WHO accredited polio laboratories in Ibadan and Maiduguri.

**Mobile Telephone Network CallerFeel strategy for polio surveillance:** Mobile Telephone Network CallerFeel is a service that allows one to express himself through short messages that appears on the caller´s phone screen when the phone line is being called. The message appears as a *pop-up notification* on the screen of the caller whenever a surveillance officer receives a call. Smileys and emoticons could also be used to make the message more exciting. Here is a sample of the message from this initiative *“Remember to report any child less than 15 years of age with sudden onset of paralysis/weakness that is floppy (i.e. not stiff...).”* This was used to create awareness in the community on case definitions of AFP, enlightened the community on COVID-19 pandemic and its preventive measures and know where to report cases of suspected AFP. Mobile telephone network is one of the mobile phone service providers in-country. Routine weekly meetings resumed virtually by the NSWG to monitor the aforementioned interventions. It should be noted that no one single independent innovative strategy was expected to reverse the declining polio surveillance indicators, but the combined cocktail of the innovations was expected to have an impact.

## Results

**Figure 2:**
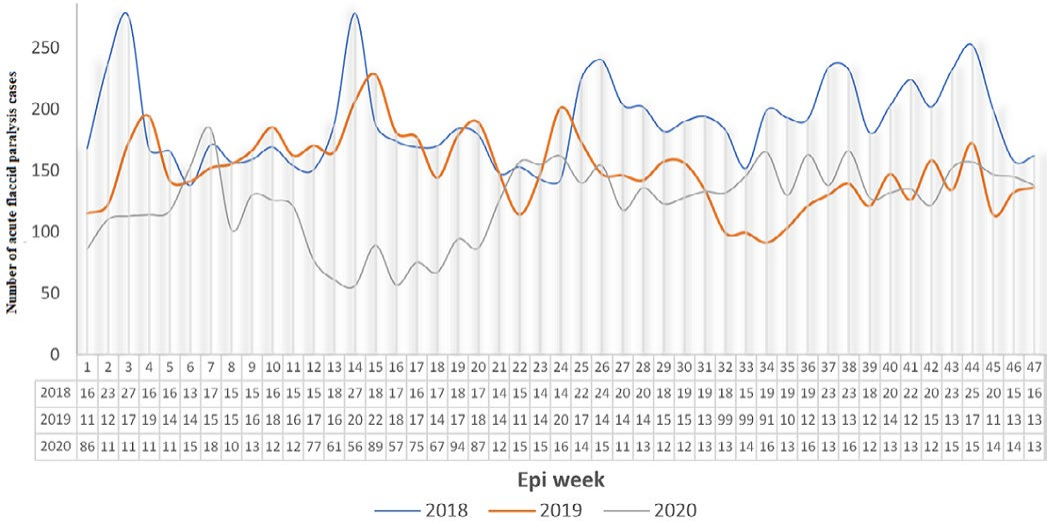
comparing the number of AFP cases reported from epidemiological week 1-47 from 2018 to 2020

**Figure 3:**
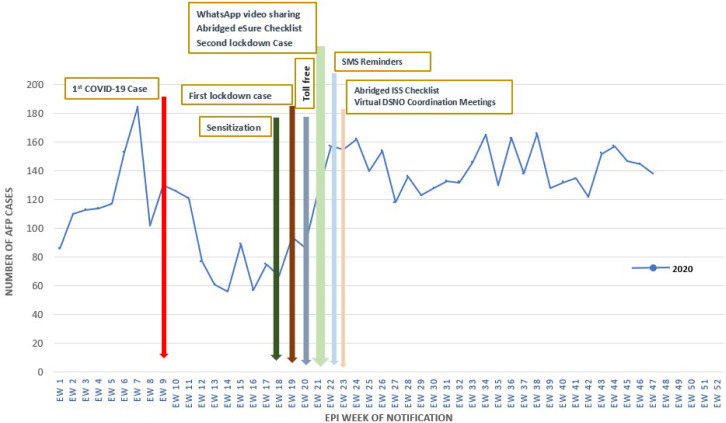
trend of AFP cases detected and reported from epidemiological week 1-47 in 2019 and 2020

**Figure 4:**
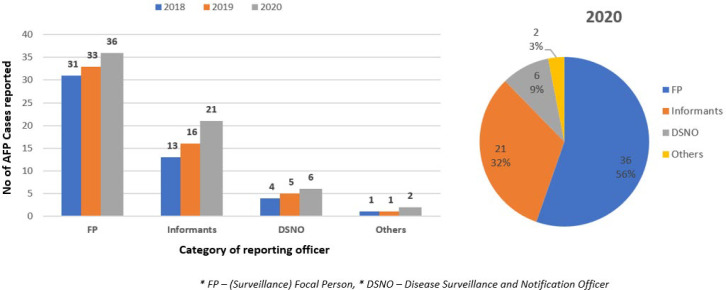
proportion of AFP cases reported by surveillance officers at the subnational levels from 2018 to October 2020

A total of 17,687 auto-visual AFP detection and reporting (AVADAR) AFP case definition videos were sent through WhatsApp every week by the 774 LGA DSNOs from week 22 - 44 while 1,689,808 AFP case definition SMS reminders were sent to the mobile number of SFP and CI from week 22 to 47 across all the 36 states and FCT. Sixty-seven percent of the DSNOs used the eSurve abridged checklist. The proportion of planned eSurve visits conducted to high priority focal sites in 2019 was 41% using the routine ODK checklist, while the proportion increased to 55% in 2020 using both routine and abridged ODK checklist. Likewise, the proportion of planned eSurve visits that were conducted increased from 50% using routine ODK in 2020 to 75% using both routine and abridged ODK checklists. Before the commencement of the interventions in weeks 1 - 20 of 2020, there was a 39.1% decline in AFP case detection compared to same period in 2019. However, for this same period in 2019, the decline was only 8.2% from that in 2018 (Figure 2). Eleven weeks into implementation of the interventions, the reduction in detection had improved from 39.1% in week 20, 2020 to 26.2% in week 31, 2020. This further reduced to 16.7% as at week 47, 2020 compared to same period in 2019 (Figure 3). There was an increase in AFP case reporting by informants, focal persons and DSNOs from 2019 through 2020: 23.8% by informants, 8.3% by focal persons and a 16.7% by DSNOs (Figure 4). Thirty-five AFP cases were detected and reported using the MTN CallerFeel strategy, while the use of a state specific toll-free number contributed to the detection and reporting of an addition 15 AFP cases.

## Discussion

This article highlights the impact of COVID-19 on polio surveillance and describes the proactive interventions by Nigeria´s polio program to enhance polio surveillance and mitigate the impact of COVID-19 on surveillance. The COVID-19 pandemic caused a marked decline in AFP detection and reporting compared to the same period in 2019. However, with initiation and implementation of innovative approaches developed and coordinated by the NSWG, the country experienced an increase in cases detection and reporting. As guided by the GPEI [21] and due to the decline in AFP case detection and reporting, the NSWG developed the surveillance contingency plan. With restrictions in movement, weekly NSWG meetings were put on hold. However, after the reports of declines in reported AFP cases, the meetings resumed. The meetings were held virtually bimonthly to observe the COVID-19 social distancing protocol as prescribed by Nigeria Center for Disease Control [24]. As at week 14, the first version of the plan was developed and shared. Second and third versions were developed and shared by weeks 18 and 30 respectively, based on feedback and easing of the lockdown. The plans were shared with the state surveillance working groups through the state epidemiologists. Weekly reports of activities carried out were received from the state surveillance group on Fridays. Feedback, after review, was provided by the NSWG on Mondays. WHO AFRO and the headquarters adopted part of the developed plan to develop regional and global guidelines.

A decline in the number of AFP cases reported was observed in 2020 just before the detection of the first COVID-19 case, and this continued to decline compared to the same period in 2019 before the pandemic. This continuous decline coincided with the period of enforcement of nationwide lockdown. A gradual increase in AFP surveillance performance was seen from week 18 with the roll-out of the first of the interventions, integrating AFP surveillance briefing with nationwide COVID-19 health worker sensitization. This increase continued with the first ease of the lockdown, the commencement of state-specific toll-free numbers, second ease of lockdown, and weekly AFP WhatsApp video messaging from weeks 18, 19, 20, 21 and 22. More cases were reported in the community by the CIs who were direct recipients of the AFP WhatsApp videos and SMS reminder messages. This was also made possible because the number of CIs was increased from 5 per ward to 10 per ward. More cases were reported in communities than the health facilities since people were not going to hospitals at the beginning of the pandemic owing to the lockdown. This scenario underscores the flexibility and adaptiveness of the polio surveillance system in particular and the resilience of the country´s polio program in general. Value of integration with other program was demonstrated, as COVID-19 response was conducted in conjunction with AFP surveillance. A good example of this is that the polio laboratory in Maiduguri was also involved in COVID-19 testing. As COVID-19 is still very much around, these approaches can be sustained due to the positive impact recorded.

**Limitations:** interpreting the contributions of these interventions has some limitations. One is the difficulties to capture contribution per innovative strategies as some were as implemented over time. This may have led to under-or over-estimation of the impact. Secondly, all strategies were not implemented to the same extent in all states, hence differential outcome.

## Conclusion

The implementation of the contingency plan reversed the declined AFP case reporting and detection, while it also reduced the exposure of surveillance personnel to the risk of COVID-19. The role of technology in improving surveillance was demonstrated as more than 70% of the interventions were information and communication technology-related.

**Recommendations:** surveillance programs should be flexible and adaptable to environmental and systemic changes. Interventions implemented during these changes need to be properly designed and debated within a team while documents are developed to monitor their impact and adjust accordingly. Lessons from the intervention and approaches can be exported to other programs like the Integrated Disease Surveillance and Response (IDSR) and RI to minimize the impact of COVID-19.

**Disclaimer:** the findings and conclusions in this report are those of the authors and do not necessarily represent the official position of the U.S. Centers for Disease Control and Prevention.

### What is known about this topic

- Certification of WPV-free status for a WHO Region requires no detection of poliovirus in any country for at least three consecutive years, as with high quality APF surveillance;
- The two main performance indicators used to determine the sensitivity of the AFP surveillance system to detect the transmission of poliovirus are the NPAFP rate and proportion of cases with stool adequacy;
- Novel coronavirus disease 2019 (COVID-19) was reported in China in December 2019 and rapidly became pandemic, preventing the implementation of usual active surveillance methods.

### What this study adds

- A rise in AFP case detection and reporting observed from the introduction of technologies to conduct remote active AFP case searches.
- Supportive supervision to focal sites increased using both the routine and abridged supervisory checklist;
- Disease surveillance and notification officers report more AFP cases than other subnational surveillance sources.

## References

[1] World Health Organization Poliomyelitis (polio).

[2] Hussain SF, Boyle P, Patel P, Sullivan R (2016). Eradicating polio in Pakistan: an analysis of the challenges and solutions to this security and health issue. Glob Health.

[3] WHO EMRO Strategy | Polio eradication initiative.

[4] Centers for Disease Control and Prevention Polio (Poliomyelitis).

[5] Nimpa MM, Razafiarivao NR, Robinson A, Fidiniaina MR, Razafindratsimandresy R, Masembe YV (2021). Efforts Towards Polio Eradication in Madagascar: 1997 to 2017. J Immunol Sci.

[6] WHO | Regional Office for Africa Polio Eradication: Africa Regional Certification Commission begins verification visit to Nigeria.

[7] WHO | Regional Office for Africa Polio.

[8] Global Polio Eradication Initiative Global Polio Surveillance Action Plan 2018-2020.

[9] Global Polio Eradication Initiative GPEI-Surveillance Indicators.

[10] Tuma JN, Wilkinson AL, Diop OM, Jorba J, Gardner T, Snider CJ (2021). Surveillance to Track Progress Toward Polio Eradication-Worldwide, 2019-2020. MMWR Morb Mortal Wkly Rep.

[11] Hamisu AW, Johnson TM, Craig K, Fiona B, Richard B, Tegegne SG (2016). Sensitivity of Acute Flaccid Paralysis Surveillance in Nigeria (2006-2015). J Infect Dis Treat.

[12] Leke RGF, King A, Pallansch MA, Tangermann RH, Mkanda P, Chunsuttiwat S (2020). Certifying the interruption of wild poliovirus transmission in the WHO African region on the turbulent journey to a polio-free world. Lancet Glob Health.

[13] Healio pediatrics "A momentous milestone": Africa declared free of wild poliovirus.

[14] The Guardian Nigeria News WHO finally declares Nigeria. African Region free of wild polio virus.

[15] World Health Organization Archived: WHO Timeline - COVID-19.

[16] World Health Organization WHO Director-General´s opening remarks at the media briefing on COVID-19 - March 11 2020.

[17] Jee Y (2020). WHO International Health Regulations Emergency Committee for the COVID-19 outbreak. Epidemiol Health.

[18] Elimian KO, Ochu CL, Ilori E, Oladejo J, Igumbor E, Steinhardt L (2020). Descriptive epidemiology of coronavirus disease 2019 in Nigeria 27 February-6 June 2020. Epidemiol Infect.

[19] Nigeria Centre for Disease Control and Prevention COVID-19.

[20] Ibrahim RL, Ajide KBello, Olatunde Julius O (2020). Easing of lockdown measures in Nigeria: Implications for the healthcare system. Health Policy Technol.

[21] Global Polio Eradication Initiative Polio eradication programme continuity: implementation in the context of the COVID-19 pandemic. Interim guide: May 2020 update v2.0.

[22] Nigeria: The African giant (1959). Round Table.

[23] WHO | Regional Office for Africa Nigeria´s Polio laboratories pass another round of accreditation exercise.

[24] Nigeria Centre for Disease Control and Prevention Federal Ministry of Health. Coronavirus Disease (COVID-19) Prevention.

